# Molecular aspects of Chikungunya virus infections in cancer patients

**DOI:** 10.1590/0074-02760210383

**Published:** 2022-04-22

**Authors:** Débora Familiar-Macedo, Bianca Ervatti Gama, Vanessa Erichsen Emmel, Gabriela Vera-Lozada, Eliana Abdelhay, Ianick Souto Martins, Rocio Hassan

**Affiliations:** 1 Instituto Nacional de Câncer, Centro de Transplante de Medula Óssea, Laboratório de Oncovirologia, Rio de Janeiro, RJ, Brasil; 2 Instituto Nacional de Câncer, Centro de Transplante de Medula Óssea, Laboratório de Células-Tronco, Rio de Janeiro, RJ, Brasil; 3 Instituto Nacional de Câncer, Comissão de Controle de Infecções Hospitalares, Rio de Janeiro, RJ, Brasil; 4 Universidade Federal Fluminense, Faculdade de Medicina, Niterói, RJ, Brasil

**Keywords:** CHIKV, oncological patients, viral load, ECSA

## Abstract

**BACKGROUND:**

Chikungunya virus (CHIKV) is an arbovirus that can cause chronic and debilitating manifestations. The first autochthonous case in Rio de Janeiro state was diagnosed in 2015, and an outbreak was declared in 2016.

**OBJECTIVE:**

The aim of this work was to evaluate CHIKV viral load in serum, plasma and urine in cancer patients to determine the best sample for diagnosis, as well as perform molecular characterisation and phylogenetic analysis of circulating strains.

**METHODS:**

Paired serum, plasma and urine collected from 31 cancer patients were tested by real-time quantitative polymerase chain reaction (qPCR) and a segment of the CHIKV E1 gene was sequenced.

**FINDINGS:**

We detected 11 CHIKV+ oncological patients. Paired samples analyses of nine patients showed a different pattern of detection. Also, a higher viral load in plasma (6.84 log_10_) and serum (6.07 log_10_) vs urine (3.76 log_10_) was found. Phylogenetic analysis and molecular characterisation revealed East/Central/Southern Africa (ECSA) genotype circulation and three amino acids substitutions (E1-K211T, E1-M269V, E1-T288I) in positive patients.

**MAIN CONCLUSION:**

The results indicate the bioequivalence of serum and plasma for CHIKV diagnosis, with urine being an important complement. ECSA genotype was circulating among patients in the period of the 2016 outbreak with K211T, M269V and T288I substitution.

Chikungunya virus (CHIKV) was first isolated in Newala District of Tanzania in 1952.^1^ The Asian lineage of CHIKV arrived in the Americas in 2013, but, in Brazil, the first autochthonous case occurred in Amapá state (September/2014) due to the introduction of the Asian genotype that circulated in the Caribbean. A few months later, the presence of the East/Central/South African (ECSA) genotype was confirmed in Bahia state (Feira de Santana).^2^ The number of cases in the country has increased exponentially and in 2016, 271,824 probable cases of chikungunya fever were registered, with 17,888 of these cases in Rio de Janeiro state.^3^

CHIKV is an arbovirus that belongs to the *Togaviridae* family, *Alphavirus* genus. The main vector is mosquitoes’ members of *Aedes* genus, *A. albopictus* and *A. aegypti*.^4^ ^,^ ^5^ It is an enveloped positive single-stranded RNA virus with approximately 11,8 kb genome that encodes four structural proteins (E1, E2, E3, C) and four non-structural proteins (nsP1, nsP2, nsP3 and nsP4).^6^

Phylogenetic analyses based on the E1 region of CHIKV genome identified three distinct genotypes: Asian, ECSA and West African genotype.^7^ In addition, a lineage descended from ECSA was identified: ECSA-derived Indian Ocean lineage (IOL).^8^ Genetic variations found in the IOL lineage have been correlated with the large epidemic that occurred in the Indian Ocean Islands and the Indian subcontinent in 2005-2006. Studies have shown that the E1-A226V, E2-I211T, E1-T98A and E2-L210Q mutations found in the IOL led to a higher affinity for the *A. albopictus* vector.^8^ ^,^ ^9^ ^,^ ^10^ Other mutations such as E1-K211E and E2-V264A have been described and correlated with an increased fitness in *A. albopictus.* ^11^

CHIKV infection has an incubation period usually of three-seven days, ranging from two-12 days,^12^ with high levels of viremia, which usually lasts four-six days after the onset of the first symptom, but it can remain for up to 12 days.^13^ ^,^ ^14^ Of those infected, 20-30% are asymptomatic,^15^ when symptomatic, they may present high fever (> 38.9ºC), polyarthralgia, skin rash, itching, eye redness, asthenia, headache and myalgia.

The impact of CHIKV infection in vulnerable populations with compromised immune systems, such as, individuals with cancer or haematopoietic stem-cell transplantation (HSCT) or even organ transplantation needs to be more discussed. Due to the different patterns of immune response in these populations, secondary infections can worsen the health and well-being of these individuals. Therefore, virological, epidemiological, immunological, and clinical studies are needed to better understand the impact of infections, like CHIKV infection, in immunocompromised populations.

So, in general terms, the aims of this study were to evaluate the best sample for diagnosis in cancer patients, check for viral load, as well as to perform molecular characterisation and phylogenetic analysis of CHIKV strains by partial E1 gene sequencing.

## SUBJECTS AND METHODS

*Study design, volunteers, and samples* - This study included thirty-one oncological patients (adults and children) in cancer treatment and regular follow-up at Instituto Nacional do Câncer José Alencar Gomes da Silva (INCA), Rio de Janeiro, Brazil. Their recruitment was performed by INCA’s Hospital infection control committees, based on clinical signs and symptoms of arboviral infection, described as: exanthema and fever, with/without pruritus, non-purulent conjunctivitis, adenomegaly, headache, neurological symptoms, myalgia, arthralgia, blood dyscrasias, limb edema, and absence of flu suggestive respiratory manifestations. Recruitment was performed within three-seven days of symptoms onset, between February and July 2016. Serum, plasma and urine samples were collected, and all cases were screened for dengue virus (DENV) serotype 1 to 4, Zika virus (ZIKV), and CHIKV by molecular diagnosis at the Oncovirology Laboratory, Bone Marrow Transplantation Centre (CEMO).

*RNA extraction, molecular diagnosis, and viral load quantification* - Viral RNA extraction was performed with the QIAamp VIRAL RNA kit (Qiagen) followed by with cDNA synthesis using 20 μL of RNA sample, with 300 ng/μL of Random hexameric primers, 40 U/μL of RNAseOUT and 200 U/μL of Superscript II Reverse Transcriptase (all from Invitrogen, Thermo Fisher Scientific). The reaction was incubated at 65ºC for 5 min and then at 25ºC for 10 min, followed by 42ºC for 1 h and 70ºC for 15 min, in a Veriti Thermal Cycler (Applied Biosystems, Thermo Fisher Scientific).

For the differential diagnosis of arboviruses infections (CHIKV, ZIKV or DENV) a real-time quantitative polymerase chain reaction (qPCR) was performed, using a TaqMan-based system (Applied Biosystems) [Supplementary data (Table I)] specific for each virus.^13^ ^,^ ^16^ ^,^ ^17^ ^,^ ^18^

The qPCR reactions were performed in duplicate using PrimeTime Std qPCR Assay (IDT) and TaqMan Universal PCR Master mix (Applied Biosystems). As well, it was included primers and probes that amplify the *HPRT1 human gene* (*assay ID Hs02800695_m1, Life Technologies*). The results were contrasted with calibration curves performed by 6 log serial dilutions of oligo DNA template (IDT) in an enriched yeast-tRNA (Ambion) Tris-EDTA buffer. The thermocycling conditions consisted of 1 cycle of 95ºC, 10 min and 50 cycles of 95ºC, 15 s and 60ºC, 1 min.

*Sequencing and phylogenetic analysis* **-** CHIKV positive samples (serum, plasma or urine with Cycle threshold ≤ 37) by the qPCR method were selected for the study of viral variants by DNA sequencing. The amplification of the segment of interest of the gene encoding the viral envelope E1 glycoprotein was performed by nested-PCR using primers that amplified 1013 bp fragments in the first reaction and 555 bp in the second reaction [Supplementary data (Table II)].^7^ ^,^ ^19^

The fragments generated were purified using Wizard SV Gel and PCR Clean-Up System (Promega) and PureLink PCR Purification Kit (Invitrogen) and sequenced using the BigDye Terminator v3.1 Cycle Sequencing (Applied Biosystems). The thermocycling conditions consisted of 40 cycles of denaturation (94ºC/10 s), annealing (50ºC/5 s) and extension (60ºC/4 min).

The sequences analysis was performed using BioEdit Sequence Alignment Editor (7.0.5.3 version) (http://www.mbio.ncsu.edu/bioedit/bioedit.htmL), sequences’ identity was performed using BLAST (http://blast.ncbi.nlm.nih.gov/Blast.cgi) and phylogenetic analysis was made using ClustalW multiple alignment with an external group (O’Nyong-nyong virus - M20303.1). See Supplementary data (Table III) for strains used. The phylogenetic tree was generated with the aid of the MEGA X Software (10.2.3 version) (http://www.megasoftware.net/) using Maximum Likelihood method and Kimura 2-parameter model, with a bootstrap of 1000 repetitions and a rate variation model allowed for some sites to be evolutionarily invariable ([+I]). Partial CHIKV genome sequences were deposited in GenBank and accession numbers were as follows: MG262507, MG262508, MG262510, MG262511, MG262512, MG262513, MG262514, MG262515, MG262516, MG262518, MG262519.

*Statistics analyses* **-** The statistical analyses were performed using One-Way ANOVA with multiple comparison using Tukey test and Kruskal-Wallis test, *p* values < 0.05 were considered significant. For analyses, GraphPad PRISM 6 (version 6.1) (GraphPad Software) was used.

*Study approval* - This study was approved by the INCA’s Ethics Committee (CAAE 53571116.4.0000.5274).

## RESULTS

*Laboratorial diagnosis of arbovirus infection and clinical characteristics of CHIKV+ oncological patients* - A total of thirty-one participants were recruited at INCA when clinical staff had a suspicion of arbovirus infection. All participants presented all selection criteria and were tested for ZIKV, CHIKV and all DENV’s serotype using qPCR reactions. We found a 35.48% positivity (11/31) for CHIKV in oncological patients and no co-infections were observed.

From the 11 CHIKV positive oncological patients, six were adults (> 18 years old) and five were under 18 years old, with a median age of 34 years (1-69 years), being 54.54% male and 45.45% female. Others clinicals features of CHIKV positive individuals are described in Table I.

**TABLE I:** Clinical features of positive Chikungunya virus (CHIKV) individuals (n = 11)

	Individuals CHIKV+	
	Oncological patients (> 18 years)^n = 6^	Oncological patients (< 18 years)^n = 5^
Age (years)		
Median	58.50	14.00
Mean ± SD	54.6 ± 13.17	11.6 ± 6.26
Gender, n(%)		
Female	4 (66.6)	1 (20)
Male	2 (33.3)	4 (80)
Oncological disease, n(%)		
Chronic myeloid leukemia (CML)	3 (50)	0 (0)
Acute myeloid leukemia (AML)	1 (16.6)	0 (0)
Hodgkin’s lymphoma	2 (33.3)	0 (0)
Glioma	0 (0)	1 (20)
Neuroblastoma III	0 (0)	1 (20)
Sarcoma^*^	0 (0)	2 (40)
PNET	0 (0)	1 (20)
Symptoms, n(%)		
Fever	6 (100)	5 (100)
Rash	6 (100)	5 (100)
Myalgia	1 (16.6)	0 (0)
Arthralgia	1 (16.6)	2 (40)

* malignant neoplasm of long bones of the lower limbs or osteosarcoma; PNET: primitive neuroectodermal tumor; SD: standard deviation.

*Viral load quantification in blood and urine specimens* - After CHIKV infection confirmation by qPCR, we decided to investigate possible differences in viral load in different samples obtained (serum, plasma and urine) in order to evaluate the best sample for CHIKV diagnosis in cancer patients.

From the 11 CHIKV positive cancer patients, the urine collection was impaired in two patients under 18 years old. So, considering nine patients with paired sample collection (serum, plasma and urine), 22.2% (2/9) had a positive result on the three specimens, 88.8% (8/9) had a positive result in serum, and 77.7% (7/9) cases had a positive result in plasma. Interestingly, just one patient had CHIKV detection only in urine (11.1%).

**Fig. 1:**
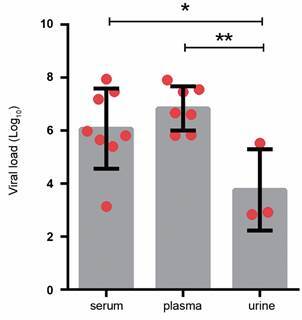
Chikungunya virus (CHIKV) viral load comparison between blood (serum and plasma) and urine specimens in oncological patients (n = 9). Data are expressed as averages ± standard deviations (scatter plot with bar). Each dot (●) represents one positive patient. Statistical analyses were performed using One-way ANOVA with multiple comparisons using Tukey test, p values < 0.05 were considered significant. Asterisks indicate significant differences (p = 0.0121).

CHIKV viraemia analyses in cancer patients showed that viral loads were significantly higher in plasma (mean 6.84 log_10,_ range 5.82 log_10_ to 7.91 log_10_) and serum (mean 6.07 log_10,_ range 3.14 log_10_ to 7.95 log_10_,), than in urine (mean 3.76 log_10,_ range 2.84 log_10_ to 5.53 log_10_) (Fig. 1).

*Phylogenetic analysis of CHIKV positive samples from oncological patients in INCA-RJ* - The phylogenetic analysis was done with representative strains (n = 11) of CHIKV detected in oncological patients from INCA (Table II).

**Fig. 2:**
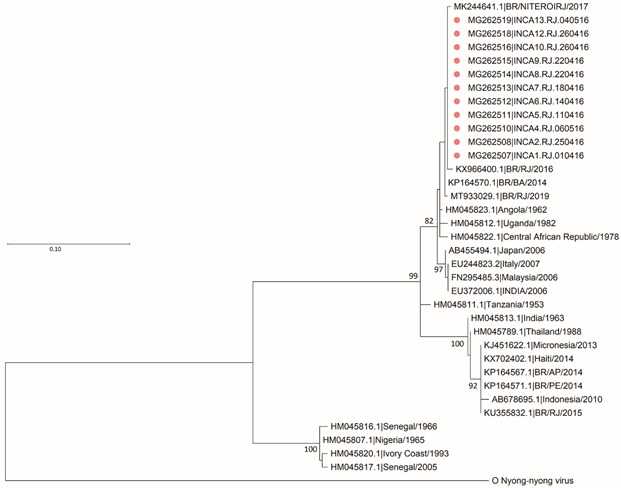
phylogenetic analysis based on 474 nucleotides recovered from the E1 gene of Chikungunya virus (CHIKV) strains identified in Rio de Janeiro (n = 11) during 2016. The analysed CHIKV sequences in this study are represented by a pink circle (●) in oncological patients (n = 11). The evolutionary history was inferred by using the Maximum Likelihood method and Kimura 2-parameter model, bootstrap of 1000 repetitions. The rate variation model allowed for some sites to be evolutionarily invariable (+I). The CHIKV strains were designated as follows: GenBank accession number/name or country/year). ECSA: East-Central-South African genotype. Evolutionary analyses were conducted in MEGA X (10.2.3 version).

Genotyping and phylogenetic reconstructions were carried out from a 474 bp fragment, revealing a similarity of 98-99% of the Rio de Janeiro circulating variants with the East/Centre/South Africa (ECSA) genotype (Fig. 2).

**Fig. 3:**
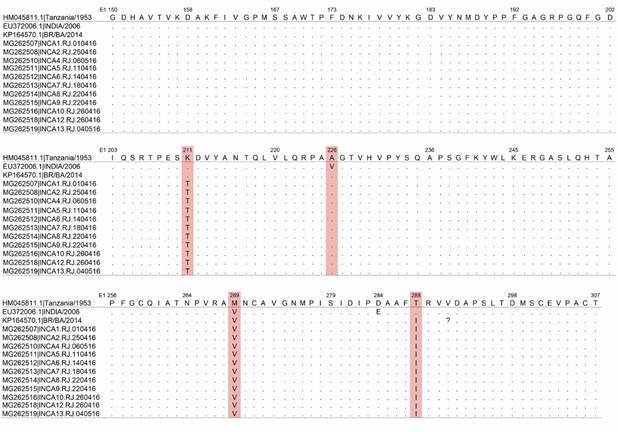
multiple amino acid alignment of the partial nucleotide sequences from Chikungunya virus (CHIKV ) E1 protein coding region showing non-synonym mutations. Amino acids positions numbered with respect to the strain Ross low-psg (GenBank Accession No. HM045811), strain DRDE-07 from India (GenBank Accession No. EU372006.1) and strain BHI3745/H804709 from Bahia state (Brazil, 2014) (GenBank Accession No. KP164570.1).

The analysis of E1 gene sequence revealed the existence of 12 synonymous mutations and three non-synonymous mutations at amino-acid level, namely K211T, M269V and T288I, when compared to HM045811 from Tanzania (1953) (Fig. 3). We also included in our analysis one isolate from India (2006) EU372006.1. We did not observe the A226V shift described for Indian isolates. All sequenced cases presented the same molecular pattern.

**TABLE II:** The Chikungunya virus (CHIKV) strains from Rio de Janeiro from oncological patients (n = 11) for partial E1 gene sequencing, in 2016, Brazil

ID samples	Year	Country, city/state	Origin of strain	GenBank accession number	Reference
INCA1.RJ.010416	2016	Brazil, Rio de Janeiro/RJ	Serum	MG262507	This study
INCA2.RJ.250416	2016	Brazil, Rio de Janeiro/RJ	Serum	MG262508	This study
INCA4.RJ.060516	2016	Brazil, Rio de Janeiro/RJ	Serum	MG262510	This study
INCA5.RJ.110416	2016	Brazil, Rio de Janeiro/RJ	Serum	MG262511	This study
INCA6.RJ.140416	2016	Brazil, Rio de Janeiro/RJ	Urine	MG262512	This study
INCA7.RJ.180416	2016	Brazil, Rio de Janeiro/RJ	Plasma	MG262513	This study
INCA8.RJ.220416	2016	Brazil, Rio de Janeiro/RJ	Plasma	MG262514	This study
INCA9.RJ.220416	2016	Brazil, Rio de Janeiro/RJ	Plasma	MG262515	This study
INCA10.RJ.260416	2016	Brazil, Rio de Janeiro/RJ	Urine	MG262516	This study
INCA12.RJ.260416	2016	Brazil, Rio de Janeiro/RJ	Serum	MG262518	This study
INCA13.RJ.040516	2016	Brazil, Rio de Janeiro/RJ	Urine	MG262519	This study

## DISCUSSION

Cancer is a situation in which patients suffer a systemic immunosuppression, that results from the particular characteristics of neoplastic diseases (i.e., some types of cancer are associated with a more severe state of immunodepression than others), the burden of disease on the body, and from the aggressiveness of current therapies like chemotherapy, radiotherapy, immunotherapy and in some cases, surgery.^20^ As well it cannot be forgotten that the heterogeneity of patient populations (past infections, malnutrition, comorbidities, cancer status etc.) may impact the treatment and can increase the risk of bacterial, fungal and viral infection. Infection is a significant cause of morbidity and mortality in these patients.^21^

CHIKV has a long history of emergence in urban transmission cycles from its enzootic and wild outbreaks in Africa, expanding to the Americas in 2013, across the Asian continent.^22^ ^,^ ^23^ In Brazil, CHIKV had two introductions, one of the Asian genotype in Amapá state and the other of the ECSA genotype in Bahia state.^2^ Driven by the presence of competent vectors (*Aedes* genus) and by an intense flow of people traveling, the number of cases in the whole country increased exponentially. Although the big burden of cases occurred in the 2016 outbreak, between 2017-2021 (until epidemiological week 29), a total of 551,393 cases were reported in Brazil and 134,209 cases in Rio de Janeiro state until today,^2^ ^,^ ^24^ ^,^ ^25^ ^,^ ^26^ ^,^ ^27^ showing that CHIKV continues to cause large numbers of cases across the country.

In this work, we were able to diagnose and evaluate the viral load of CHIKV infection in cancer patients who were being followed up at INCA in Rio de Janeiro state. A total of 11/31 oncological patients were CHIKV positive by qPCR at least in one sample (plasma, serum or urine). Indeed, we believe that this number is underestimated, because samples collection was made at the time of CHIKV outbreak flourishing, and so a higher positive rate was expected. The goal of cancer treatment is based on selective killing of the cancer cells. Chemotherapy, for example, is the most common treatment used that is supposed to target just cancer cells, nonetheless, normal cells suffer damage too, and these damages lead to a range of manifestations and side-effects. Among them, headache, fatigue, weakness, hair loss, nausea, vomiting, diarrhea, abdominal cramps, fever, skin rash^28^ can mask signs and symptoms of viral infections. Hence, when the patient is in the cycle chemotherapy and may have a viral infection, such as CHIKV infection, one could first suspect the treatment side-effects and afterwards the infections. Consequently, some patients may not have looked for medical care and viral diagnosis not made.

Some viruses, like CHIKV, can cause high levels of viraemia in humans in acute phase.^29^ ^,^ ^30^ In blood, CHIKV can reach up 10^8^ or 10^9^ genome copies/mL.^31^ ^,^ ^32^ ^,^ ^33^ A case of series has analysed solid organ transplantation (SOT) recipients with CHIKV infection. Rosso et al. has observed that 80% of SOT patients presented high viral load (> 10^6^ copies/mL) and none of them developed graft rejection, chronic inflammatory manifestations or fatal cases after CHIKV infection,^34^ indicating that CHIKV infection may not have a negative impact on SOT patients. On other hand, another study suggested a negative impact on immune response in HIV-positive patients (elite controller) that live in a CHIKV endemic area in Martinique.^35^ Therefore, more studies in immunosuppressed individuals are needed.

In our study, we proposed to quantify CHIKV viral load in cancer patients in blood and urine. Although the positive final dataset is small, we observed that our patients present a high viral load in plasma (mean 6.84 log_10_) and serum (mean 6.07 log_10_) similar to SOT patients described in a case of series by Rosso and collaborators.^34^ Also, we were able to detect and quantify viral load in urine of 33.33% (3/9) patients (mean 3.76 log_10_) that was significantly lower than the viral load present in plasma. Of note, our detection rate in urine is much higher than that reported in French Polynesia outbreak.^36^

Studies have been reporting the importance of using urine in arbovirus diagnosis.^37^ Bandeira and collaborators in a case-report study detected CHIKV RNA in urine and semen for an extended period (30 days).^38^ Moreover, another study has described that DENV RNA detection rate in urine, on acute phase, was 25% (2/8) on days 0 to 3 and 32% (7/22) on days 4 to 5. As the days went by, the detection rate increased until day 11 after the first symptom appearance and started to decline until the last day of detection (day 16). The authors suggested that urine can be an ally to molecular diagnosis when viremia disappears.^39^ Herein, we did not evaluate the cancer patients in multiples collections and different days after the first CHIKV detection, so, we may speculate that the detection rate in urine could rise if we had multiple collections some days later.

Furthermore, we found a similarity of 98-99% of the circulating variants in oncological patients from Rio de Janeiro state with ECSA. Other studies showed the circulation of the ECSA genotype in the state of Rio de Janeiro in 2016,^40^ ^,^ ^41^ ^,^ ^42^ as the present study shows, but also the permanence of this strain until today.^43^

In addition, we made molecular characterisation of CHIKV strains. We found 12 synonym mutations and three non-synonym mutations (E1-K211T, E1-M269V and E1-T288I). Souza et al. showed two amino acids substitutions (E1-K211T and E1-V156A) which are exclusive to the CHIKV strains obtained also during the 2016 epidemic in Rio de Janeiro (Brazil).^41^ More studies are needed to evaluate the consequences of these mutations on viral replication, in human immune response and in vector fitness (*A. albopictus* and *A. aegypti)*. Knowing the vector competence of each species is important to understand the transmissibility potential of mosquitos, and therefore it is important to evaluate the potential amino acid substitution and its impact in virus replication to aid epidemiological studies and epidemics previsions.^44^

We conclude that CHIKV genome detection can be performed with equal efficiency for both serum and plasma. Viral loads found in the serum and plasma of oncological patients infected with CHIKV were greater than those found in the urine. Although we are not able to discard a late virus excretion, the detection of the single case with virus detection only in urine suggests that this compartment may be an important complement to diagnostics. Finally, phylogenetic analyses had shown that the circulating genotype among cancer patients in 2016 outbreak belonged to the ECSA genotype, carrying K211T, M269V and T288I substitutions in E1-CHIKV genome.
