# Supplementary material for: Molecular aspects of Chikungunya virus infections in cancer patients
Source: Mem Inst Oswaldo Cruz. 2022 Apr 22;117:e210383. doi: 10.1590/0074-02760210383 (PMC9037814; doi:10.1590/0074-02760210383)
Supplement: Supplementary file 1 [file 1678-8060-mioc-117-e210383-s.pdf]

TABLE I

Primers and probes used to differential arbovirus diagnosis

References	Virus	Primer/Probe	Sequence (5'-3')	Position	Product (bp)
Waggoner and Pinsky, 2016	Zika virus	Zika 4481	CTGTGGCATGAACCCAATAG	4434-4453	90
		Zika 4552c	ATCCCATAGAGCACCACTCC	4524-4505	
		Zika 4507c-FAM	CCACGCTCCAGCTGCAAAGG	4479-4460	
Lanciotti, 2008	Zika virus	ZIKV 1086	CCGCTGCCCAACACAAG	1086-1102	76
		ZIKV 1162c	CCACTAACGTTCTTTTGCAGACAT	1162-1139	
		ZIKV 1107-FAM	AGCCTACCTTGACAAGCAGTCAGACACTCAA	1107-1137	
Santiago, 2013	Dengue 1	D1-F	CAAAAGGAAGTCGYGCAATA	8936-8955	112
		D1-R	CTGAGTGAATTCTCTCTGCTRAAC	9023-9047	
		D1-FAM	CATGTGGYTGGGAGCRCGC	8961-8979	
	Dengue 2	D2-F	CAGGCTATGGCACYGTCACGAT	1426-1447	78
		D2-R	CCATYTGCAGCARCACCATCTC	1482-1504	
		D2-VIC	CTCYCCRAGAACGGGCCTCGACTTCAA	1454-1480	
	Dengue 3	D3-F	GGACTRGACACACGCACCCA	701-720	74
		D3-R	CATGTCTCTACCTTCTCGACTTGYCT	749-775	
		D3-TX red	ACCTGGATGTCGGCTGAAGGAGCTTG	722-747	
	Dengue 4	D4-F	TTGTCCTAATGATGCTRGTCG	884-904	89
		D4-R	TCCACCYGAGACTCCTTCCA	953-973	
		D4-CY5	TYCCTACYCCTACGCATCGCATTCCG	939-965	
Lanciotti, 2007	Chikungunya virus	CHIKV 6856	TCACTCCCTGTTGGACTTGATAGA	6856-6879	125
		CHIKV 6981	TTGACGAACAGAGTTAGGAACATACC	6981-6956	
		CHIKV 6919-FAM	AGGTACGCGCTTCAAGTTCGGCG	6919-6941	

TABLE II

Primers sets used for evaluation of mutations and phylogenetic analysis

References	Primers	Sequence (5'-3')	Position	Product (bp)
Santhosh et al. 2008	E1-primary-F	ACAAAACCGTCATCCCGTCTC	10145-11158	1013
	E1-primary-R	TGACTATGTGGTCCTTCGGAGG		
Niyas et al. 2010	E1-F1	GCTCCGCGTCCTTTACC	10389-10943	555
	E1-R1	ATGGCGACGCCCCCAAAGTC		

CC BY

TABLE III

Representative sequences of three different Chikungunya virus (CHIKV) genotypes (Asian, East-Central-South African (ECSA), and West African) used for phylogenetic analysis

Genotype	ID samples	Year	Country, city/state	GenBank accession number
ECSA	BR|NiteroiRJ|2017	2017	Brazil, Niteroi,/RJ	MK244641.1
	BR|RJ|2019	2019	Brazil, Duque de Caxias/RJ	MT933029.1
	BR|PB|2016	2016	Brazil, João Pessoa/PB	KY704955.1
	BR|RJ|2016	2016	Brazil, Rio de Janeiro/RJ	KX966400.1
	Angola|1962	1962	Angola	HM045823.1
	Uganda|1982	1982	Uganda	HM045812.1
	Central African Republic|1978	1978	Central African Republic	HM045822.1
	BR|BA|2014	2014	Brazil, Bahia/BA	KP164570.1
	Tanzania|1953	1953	Tanzania	HM045811.1
	Japan|2006	2006	Japan	AB455494
	Italy|2007	2007	Italy	EU244823.2
Asian	Brazil|AP|2014	2014	Brazil, Oiapoque/AP	KP164567.1
	BR|PE|2014	2014	Brazil, Recife/PE	KP164571.1
	BR|RJ|2015	2015	Brazil, Rio de Janeiro/RJ	KU355832.1
	Haiti|2014	2014	Haiti	KX702402.1
	Indonesia|2010	2010	Indonesia	AB678695.1
	Malaysia|2006	2006	Malaysia	FN295485.3
	Thailand|1988	1988	Thailand	HM045789.1
	India|1963	1963	India	HM045813.1
	Micronesia|2013	2013	Micronesia	KJ451622.1
West African	Nigeria|1965	1965	Nigeria	HM045807.1
	Senegal|2005	2005	Senegal	HM045817.1
	Ivory Coast|1993	1993	Ivory Coast	HM045820.1
	Senegal|1966	1966	Senegal	HM045816
